# The effect of physical fatigue on the performance of soccer players: A systematic review

**DOI:** 10.1371/journal.pone.0270099

**Published:** 2022-07-14

**Authors:** Felipe Dambroz, Filipe Manuel Clemente, Israel Teoldo

**Affiliations:** 1 Department of Physical Education, Centre of Research and Studies in Soccer (NUPEF), Universidade Federal de Viçosa, Viçosa, Brazil; 2 Escola Superior Desporto e Lazer, Instituto Politécnico de Viana do Castelo, Rua Escola Industrial e Comercial de Nun’Álvares, Viana do Castelo, Portugal; 3 Research Center in Sports Performance, Recreation, Innovation and Technology (SPRINT), Melgaço, Portugal; 4 Instituto de Telecomunicações, Delegação da Covilhã, Lisboa, Portugal; University of L’Aquila Department of Clinical Sciences and Applied Biotechnology: Universita degli Studi dell’Aquila Dipartimento di Scienze Cliniche Applicate e Biotecnologiche, ITALY

## Abstract

This study aimed to carry out a systematic review to analyze, describe and discuss the effect of physical fatigue on the performance of soccer players. For this systematic review, searches were performed in Pubmed, Web of Science and SPORTDiscus electronic database until October 3, 2020, following the guidelines of PRISMA. A total of 12 articles met the inclusion criteria: i) healthy soccer players from any age group, competitive level or sex; ii) exposure to physical fatigue; iii) pre and post-physical fatigue conditions; iv) players’ cognitive, technical, physical and tactical performances and v) no restrictions regarding the study design. The results section was organized in four main dimensions: cognitive, technical, physical and tactical. Studies on cognitive performance have shown divergent results, varying according to the cognitive task employed and the physical protocol used. Regarding technical performance, negative effects of physical fatigue were found on the technical fundamentals of the pass, dribble and kick. With regard to physical performance, studies have shown a reduction in sprint capacity and distances covered at high velocity. Finally, the only study that analyzed the tactical performance in the field showed an increase in the team’s collective tactical behavior, but did not include analysis of the players’ individual tactical actions. In summary, the results of the analyzed studies show that the effect of physical fatigue on cognitive performance is inconclusive and that technical and physical performance are negatively affected. Regarding tactical performance, there is a lack of information on the topic in the current literature.

## Introduction

Soccer is a game of cooperation and opposition between players and teams, which results in a complex and unpredictable dynamic [1,2]. In this way, the positioning and the action of the players around the playing field depend on the organization of teammates, opponents and the information available in the environment, which are presented dynamically and throughout the game [3]. Thus, the different situations and contexts of the game demand from players specific behaviors and a high number of decision-making, in order to solve the problems presented during the game [2,4].

In this context, for the maintenance of sports performance, well-developed tactical, technical, physical, and cognitive skills are increasingly being demanded from players. Additionally, the competitive calendar of soccer teams through the seasons presents a high load of training and games [5]. For example, some teams play multiple games with a minimal recovery period between successive matches, besides interchanging these games with training sections and long trips [6]. Thus, if load control is not well-performed, it could affect the performance, impact in-game quality, and increase the number of injures [7,8].

During official matches and training sections, players are exposed to varied physical demands, including running, jumping, sprints, accelerations, decelerations, and constant changes in direction [9], which results in physiological alterations. Likewise, players are required to have a high cognitive effort for maintaining high attention levels, anticipating opponents’ actions and making decisions in a restricted space and time combined with constant pressure from the environment (e.g. game situations, opponents, fans, etc.) [10,11]. A consequence of these demands is fatigue, a sensation of tiredness and weakness [12], or failure to maintain the required or expected force during and following exercise [13], which is induced by multi-factorial phenomena.

Fatigue occurs when the degree of effort exceeds the player’s ability to support the task [12,14]. In soccer, the players temporarily experience fatigue during the game and towards the end of the matches [14,15]. As a consequence, fatigue has been considered as a potential factor influencing the performance of soccer players. Over the past few years, fatigue has been investigated from physical and physiological perspectives [15,16]. Following this and considering a practical perspective, acute physical fatigue is characterized by changes in the organism manifested by neuromuscular changes and metabolic and psychometric responses [14,17].

Physical exercise causes micro cellular injuries in muscle tissue. Thus, when a person is physically fatigued, the muscle recovery process is impaired, resulting in a decrease in the efficiency of the recruitment of muscle fibers that control movement, in addition to increasing the likelihood of injuries [14,18]. Thus, if the player is physically worn out, the greater the need for movement adjustments and the less qualified these actions tend to be during the game, since acute physical fatigue causes neurophysiological changes that affect the central nervous system [19] and impair movement on the motor and physical plane [20,21]. Given these changes, researchers have identified that physical fatigue leads to a lower frequency of movements such as accelerations and sprints [22], decreases the distance covered and the velocity of actions [23], reduces the efficiency of passes and shooting [24], and increases the incidence of goals in the final minutes of a match [25].

Based on these, the studies on acute physical fatigue, especially in the soccer context, attribute negative effects of it on physical and technical performances [15]. Concerning cognitive and tactical aspects, it has been argued that few researchers have been paying attention on these aspects, even though the players’ performance is conceived based on the interaction of cognitive, technical, physical, and tactical elements [2]. Additionally, the limitations in evaluating players’ performance could be contributing to misconception conclusions and difficulties in applying the results in practical terms [26]. Therefore, understanding the effects of physical fatigue on the players’ performance from the cognitive, technical, physical, and tactical perspectives is necessary for the development of strategies that can help the players to face this condition during a match, as well as for reduce the players’ susceptibility to injury.

Given the relevance of this topic, the aim of this article is to systematically review the studies available in the literature, in order to verify the effect of physical fatigue on the sports performance of soccer players. By doing that, it is expected to provide information for understanding the individual and collective performance of players subjected to physical fatigue, besides identifying possibilities for future investigations and interventions, considering physical fatigue as a conditioning factor of the cognitive, technical, physical, and tactical performance of soccer players.

## Materials and methods

This systematic review was carried out according to the recommendations of the PRISMA method—(Preferred Reporting Items for Systematic Reviews and Meta-Analyzes) [27]. The protocol was registered with the International Platform of Registered Systematic Review and Meta-Analysis Protocols with the number 202150054 and the DOI number 10.37766/inplasy2021.5.0054. The criteria used for the inclusion of the studies followed the guidelines of the PICOS strategy (Table 1).

**Table 1:** Definition of PICOS for inclusion and exclusion of articles.

Parameter	Definition
Population	Healthy soccer players from any age group, competitive level or sex.
Intervention	Exposure to physical fatigue
Comparison	Pre- and post-physical fatigue conditions
Outcomes	Players’ cognitive, technical, physical and tactical performances
Study Design	No restrictions regarding the study design

PICOS: Population, Intervention, Comparison, Outcomes and Study design.

### Databases and search strategies

Searches for scientific articles were carried out from October 01 to 03, 2020 in the electronic databases *Web of Science* (from 1945 to 2020), PubMed and *SPORTDiscus*. The terms used for the searches were: (soccer OR football) AND (“neuromuscular fatigue” OR “muscular fatigue” OR “physical fatigue” OR fatigue OR “physical exertion” OR “physical load”) AND (performance). These descriptors were combined with the use of the Boolean operators "AND" and "OR". Additionally, the reference lists of the included studies retrieve were manually searched to identify potentially eligible studies not captured by the electronic searches.

### Screening and eligibility of studies

The initial screening was based on reading the title and summary of the articles. Duplicate articles between databases were excluded. Next, some articles were selected to read the full text in order to verify that they met all the inclusion criteria described by the PICOS strategy, as shown in Table 1. The eligibility criteria adopted were: i) articles published in international peer-reviewed journals; ii) validity and reliability of the instruments used in studies established and published in scientific journals; iii) research conducted with human beings; iv) articles related only to soccer; v) published in English, Portuguese or Spanish. In addition, the exclusion criteria adopted were: i) review studies; ii) works published in conference proceedings (abstracts); iii) articles on ergogenic resources and nutritional interventions; iv) studies on other sports.

### Data extraction

All extracted data were registered in a form prepared in Microsoft Excel (Microsoft Corporation, Readmon, WA, USA), adapted from the Cochrane Consumers and Communication Review Group’s [28]. That form was used to assess inclusion requirements and subsequently tested for all selected studies. The process was independently conducted by the two authors (FRD and FM). In case of disagreements regarding the selection of articles, a third reviewer (FSLC) was consulted to define whether or not to include the article in this study. Full text articles excluded, with reasons, were recorded. All the records were stored in the sheet.

### Data items

The selected articles were classified according to their methodological quality aiming to understand the data reliability. The methods used to evaluate the performance outcomes were extracted from the studies, which evaluated pre- and post-physical fatigue conditions. Overall, for the technical performance, the studies evaluated the player’s ability to execute technical fundamentals (e.g., pass, kick and dribble). Regarding the cognitive performance, the player’s efficiency in decision-making (e.g., quality and response time) and perception skills (e.g., reaction time, attention and visual search) were the variables analyzed by the studies. The results on physical performance were obtained through the sprint capacity and the distance covered by the players. Finally, the tactical performance was evaluated based on the team’s collective tactical behavior (e.g., synchronization movements and player dispersion). The physical protocols were extracted from the articles to obtain information about the physical fatigue to which the players were submitted. To understand the physical state of the players, the physical-physiological variables used by the articles were considered. Additionally, the following information was extracted from the selected articles: authors and year of publication; goals; sample description; instruments; analysis/data source; and results.

### Methodological assessment

An adapted version of risk-of-bias quality form (16 items) was used to evaluate the quality of the studies [29]. The articles were evaluated according to the items presented in Table 2. Each item was evaluated using a binary scale (1—meets the criteria; 0—does not meet the criteria). The articles’ quality was individually expressed through a final score corresponding to the sum of the scores that met the criteria (1) divided by the total number of scored items [16] (Table 3). The articles were classified based on the final score: low methodological quality (≤ 50%), good methodological quality (between 51% and 75%), and excellent methodological quality (> 75%), as defined in previous studies [30,31].

**Table 2:** Inclusion and exclusion criteria.

Item	Inclusion Criteria	Exclusion Criteria
Population	Healthy soccer players from any age group, competitive level or sex	Injury; illness or other clinical condition and studies on other sports.
Intervention/ exposure	Exposure to physical fatigue	Exposure to ergogenic resources and nutritional interventions
Comparator	Pre- and post-physical fatigue conditions	Any test realized during physical protocol
Outcome	Players’ cognitive, technical, physical and tactical performances	Strength measurement, biomechenical aspect or metabolic response
Study design	No restrictions regarding the study design	-
Additional criteria	Articles published in international peer-reviewed journals	Review studies and works published in conference proceedings (abstracts)

**Table 3:** Methodological quality assessment scoring system.

Question		Answer	Score
Q1	study purpose stated clearly?	Yes = 1; No = 0	0–1
Q2	relevant background literature reviewed?	Yes = 1; No = 0	0–1
Q3	design appropriate for the research question?	Yes = 1; No = 0	0–1
Q4	sample described in detail?	Yes = 1; No = 0	0–1
Q5	sample size justified?	Yes = 1; No = 0	0–1
Q6	informed consent obtained?	Yes = 1; No = 0	0–1
Q7	outcome measures reliable?	Yes = 1; No = 0	0–1
Q8	outcome measures valid?	Yes = 1; No = 0	0–1
Q9	method described in detail?	Yes = 1; No = 0	0–1
Q10	results reported in terms of statistical significance?	Yes = 1; No = 0	0–1
Q11	analysis methods appropriate?	Yes = 1; No = 0	0–1
Q12	importance for the practice reported?	Yes = 1; No = 0	0–1
Q13	any drop-outs reported?	Yes = 1; No = 0	0–1
Q14	conclusions appropriate given the study methods?	Yes = 1; No = 0	0–1
Q15	implications for practice given the results of the study?	Yes = 1; No = 0	0–1
Q16	limitations of the study acknowledged and described by the authors?	Yes = 1; No = 0	0–1
Total			0–16

Adapted from Law and colleagues (1998).

## Results

The flowchart of the article selection process was developed according to the PRISMA guidelines and is characterized in the figure below.

### Study identification and selection

**Fig 1:**
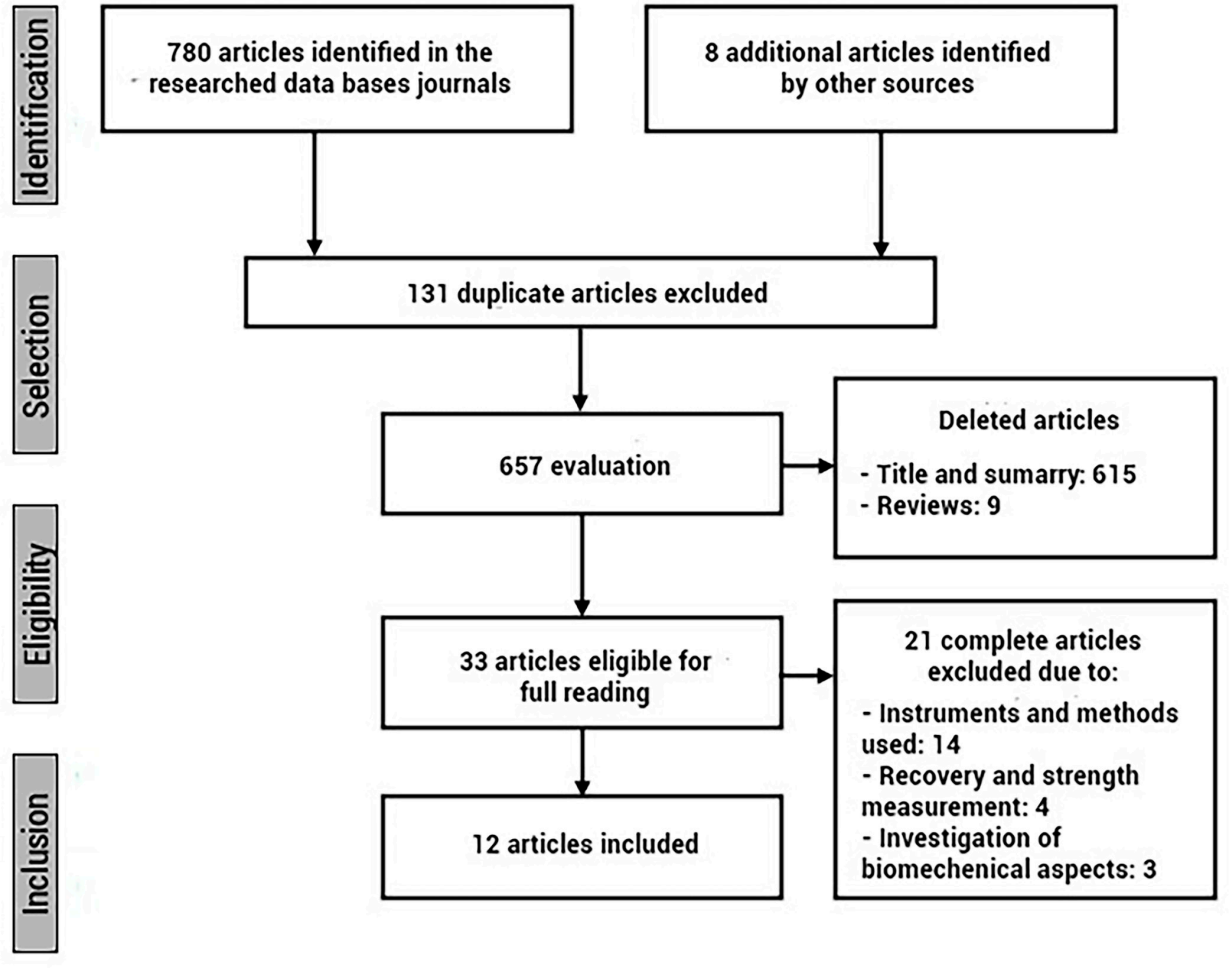
Flowchart of the review process.

The initial search identified a total of 788 articles. These studies were then exported to reference manager software (Mendeley Desktop version 1.19.5 for Windows). Any duplicates (131 references) were subsequently removed either automatically or manually. The remaining 657 articles were screened for relevance based on titles and abstracts, resulting in the removal of a further 624 studies. Following the screening procedure, 146 articles were selected for in depth reading and analysis. After reading full texts, a further 21 studies were excluded due to not meeting the eligibility criteria with instruments and methods used (n = 14), recovery and strength measurement (n = 4) or investigation of biomechanical aspects (n = 3). At the end of the screening procedure, 12 studies were included in the qualitative and quantitative synthesis (Fig 1).

### Data extraction and synthesis

#### Quality assessment

The main results concerning the studies’ quality according to the distributed scores are shown in Table 4. The average score of the methodological quality analysis of the 12 articles included in this review was 77.80%. In addition, seven articles (58.34%) were classified as excellent methodological quality (> 75%), four articles (33.33%) as good methodological quality (between 51% and 75%), and only one article (8.33%) as low methodological quality (≤ 50%).

**Table 4:** Assessment of studies’ quality according to the pre-established methodological quality scoring system.

Number	Author	Year	Q1	Q2	Q3	Q4	Q5	Q6	Q7	Q8	Q9	Q10	Q11	Q12	Q13	Q14	Q15	Q16	Percentual	Classification
1	McMorris e Graydon	1996	1	1	0	0	0	1	0	0	1	1	1	0	0	1	0	1	50.00%	LOW
2	Lemmink e Visscher	2005	1	1	1	1	0	1	1	1	1	1	1	0	0	1	1	1	81.25%	EXCELLENT
3	Rampinini e colleagues	2008	1	1	1	1	0	1	1	1	1	1	1	1	0	1	1	1	87.50%	EXCELLENT
4	Fontana e colleagues	2009	1	1	1	1	0	1	1	1	1	1	1	0	0	1	0	1	75.00%	GOOD
5	Stone e Oliver	2009	1	1	1	1	0	1	0	1	1	1	1	1	0	1	1	1	81.25%	EXCELLENT
6	Small e colleagues	2009	1	1	1	1	0	1	1	1	1	1	1	1	0	1	1	1	87.50%	EXCELLENT
7	Russell e colleagues	2011	1	1	1	1	0	1	1	1	1	1	1	1	0	1	0	1	81.25%	EXCELLENT
8	Ferraz e colleagues	2012	1	1	1	1	0	1	1	0	1	1	1	0	0	1	0	1	68.75%	GOOD
9	Casanova e colleagues	2013	1	1	1	1	0	1	1	1	1	1	1	1	0	1	1	1	87.50%	EXCELLENT
10	Frýbort e colleagues	2016	1	1	1	1	0	1	1	0	1	1	1	0	0	1	0	1	68.75%	GOOD
11	Coutinho e colleagues	2018	1	1	1	1	0	1	1	1	1	1	1	1	0	1	1	1	87.50%	EXCELLENT
12	Barte e colleagues	2020	1	1	1	1	0	1	1	0	1	1	1	0	0	1	1	0	68.75%	GOOD

#### Data extraction and synthesis

After the search and screening processes, twelve articles were selected to compose this systematic review. From a chronological point of view, the selected studies were published between 1996 and 2018. Table 5 presents the summary of the articles included in the review.

**Table 5:** Summary of studies included in the systematic review.

Authors	Objectives	Sample description	Instruments	Analysis/data sources	Results
McMorris and Graydon (1996) [34]	To verify the effect of physical exercise on soccer players’ decision making	20 soccer players (10 experienced and 10 inexperienced)	▪ Exercise on the intensity cycle ergometer 70% and 100% VO^2^max ▪ 10 offensive situations (picture of situation 6 vs. 6)	▪ quality of decision making ▪ decision-making time ▪ correct decision-making time	General ↔ quality of decision making ↓ correct decision-making time ↓ decision making time Experienced ↔ quality of decision making ↓ general decision-making time ↓ correct decision-making time Inexperienced ↔ quality of decision making ↔ correct decision-making time ↓ VO2 decision making time 100%
Lemmink and Visscher (2005) [33]	To check the effect of intermittent exercise on response time	16 recreational soccer players (20.9 ± 2.0 years)	▪ Exercise on the cycle ergometer 3 blocks– 8 minutes Alternating intensity: • high (40 seconds—4 W/kg) • low (20 seconds—75 W) ▪ Vienna system - Multiple choice task	▪ HR ▪ [La] ▪ median reaction time ▪ number of correct reactions ▪ number of incorrect reactions ▪ number of reactions omitted	↔ FC; ↔ [La] ↔ median reaction time ↔ number of correct reactions ↔ incorrect and omitted reactions
Rampinini and collaborators (2008) [35]	To verify if the fatigue accumulated during the game or generated by short sessions of high intensity intermittent activities affect the passing ability.	16 soccer players (17.6 ± 0.5 years)	▪ Soccer game lasting 90 minutes ▪ 10 consecutive 40 meter back and forth runs (5 to 16 km/h and 5 to 19 km/h) 25 seconds of recovery ▪ Loughborough Soccer Passing Test (LSPT)	▪ RPE ▪ HR ▪ [La] ▪ pass accuracy ▪ LSPT performance ▪ LSPT penalty ▪ correlation between distance covered in the Yo-Yo Intermittent Recovery Test Level 1 test and pass performance	During the game ↓ pass accuracy ↓ LSPT performance ↑ LSPT penalty ↑ RPE ↓ HR and [La] High intensity exercise Negative correlation between performance in the Yo-Yo Test with passing accuracy, performance and penalties in the LSPT
Fontana and collaborators (2009) [38]	To verify the effect of exercise intensity on the decision making of experienced and inexperienced soccer players.	32 soccer players (16 experienced—21.13 ± 1.62 years; 16 inexperienced 19.54 ± 1.14 years).	▪ Exercise on the treadmill with different intensities: 40%, 60% and 80% VO^2^max ▪ Fontana-Gallagher Soccer Decision-Making Instrument	▪ quality in decision making ▪ decision-making time	General ↔ quality of decision making ↓ decision-making time at intensities 60% and 80% of both groups. Experienced ↑ quality in decision making ↑ faster decision making
Stone e Oliver (2009) [40]	To investigate the effect of fatigue developed during intermittent high intensity exercises on the technical basis of dribbling and kicking	9 semi-professional soccer players (20.7 ± 1.4 years)	▪ Loughborough Intermittent Shuttle Test (adapted) 45 minutes ▪ Slalom dribble test e Loughborough Soccer Shooting Test–LSST	▪ HR ▪ sprints ▪ dribbling time ▪ shooting score	↑ HR; ↔ sprints ↑ dribbling time ↓ shooting score
Small and collaborators (2009) [41]	To investigate the effect of a soccer-specific multi-directional fatigue protocol in sprint capacity, sprint kinematics and risk of injury to the posterior thigh	9 semi-professional soccer players (21.3 ± 2.9 years)	▪ SAF T^90^ physical test ▪ 10 meter sprint every 15 minutes during the physical test	▪ sprint capacity ▪ sprint kinematics ▪ hamstring muscle length;	↓ sprint Capacity ↓ sprint Kinematics ↑ risk of injury to the posterior thigh
Russel and collaborators (2011) [39]	To verify the effect of fatigue induced by a soccer match simulation protocol on technical performance	15 young soccer players (18.1 ± 0.9 years)	▪ Soccer game simulation test– 90 minutes (Nicholas et al., 2000) ▪ Pass and shooting skill test	▪ [La] ▪ HR ▪ sprint ▪ pass accuracy/shooting; ▪ % pass/shooting success; ▪ average speed of the ball in the pass/shooting.	↑ [La]; *↓*Sprint; ↑HR ↓ shooting accuracy ↔ % of success of the shooting; ↔ average shooting speed ↔ accuracy, % pass success ↓ average pass speed ↔ accuracy, % success and average dribbling speed
Ferraz and collaborators (2012) [42]	To investigate the influence of physical fatigue on the maximum speed of the ball in soccer shooting.	10 amateur soccer players (27.3 ± 5.25)	▪ Specific circuit 5 repetitions– 90 seconds ▪ To shooting the ball with maximum force on the target (1x1 at 1 meters high in the middle of the goal 3x2 meters) distant at 7 meters .	▪ HR ▪ RPE ▪ maximum ball speed ▪ average ball speed ▪ distance covered	↑ HR; ↑ RPE; ↑ [La] ↓ maximum and average speed of the ball in soccer shooting ↔ distance covered
Casanova and collaborators (2013)	To verify whether prolonged intermittent physical exercise has an effect on visual search, verbal reporting and the ability to anticipate of soccer players at different levels.	16 soccer players (8 elite 24.6 ± 3.9 years; 8 sub-elite bass 26.3–2.9 years).	▪ Intermittent exercise protocol specific to soccer on the treadmill (Drust et al. 2000). ▪ 40 scenes from different offensive sequences divided into 4 blocks	▪ HR ▪ [La] ▪ % accuracy anticipation ▪ fixation time ▪ fixation number ▪ fixation location ▪ cognitive ▪ evaluative ▪ evaluative and planned type	General ↑ HR; ↑[La] ↓ % accuracy anticipation Elite ↓ % accuracy anticipation ↓ fixing number ↔ fixing time ↓ duration of fixation ↓ attachment location ↓ in-depth planning statements Sub-elite ↓ % accuracy anticipation ↑ fixation number ↑ fixation time ↑ short-term fixation ↑ fixation location ↑ fixation time on ball carrier ↓ fixation time on the ball ↓ number of cognitive statements
Frýbort and collaborators (2016)	To analyze the relationship between the variation of the intensity of the exercise and the time of visual-motor response and the accuracy of the motor response in an offensive game situation in soccer.	42 semi-professional soccer players (18.0 ± 0.9 years)	▪ Treadmill protocol Exercise intensity: 1. Inactive for 4 minutes; 2. Aerobic—4 minutes running on the treadmill (12–14.5 km/h) 3. Intermittent—4 minutes running (17–19 km/h) and walking on the treadmill; 4. Anaerobic—30 Seconds running on the treadmill (18–20 km/h) ▪ 16 offensive sequence scenes	▪ correct motor response ▪ response time visual-motor	↔ correct motor response ↑ visual-motor response time after moderate aerobic exercise compared to anaerobic exercise.
Coutinho and collaborators (2018)	To verify the effect of mental and muscular fatigue on the physical performance and on the tactical behavior of soccer players	10 amateur soccer players (13.7 ± 0.5 years)	▪ RCOD Protocol (Beckett, Schneiker, Wallman, Dawson and Guelfi, 2009) ▪ 3 x 6’ game reduced in 5 vs 5 configuration	▪ RPE ▪ distance covered high intensity ▪ distance covered moderate intensity ▪ distance covered low intensity ▪ acceleration ▪ deceleration ▪ distance between the dyads ▪ distance dyads ▪ longitudinal synchronization ▪ side synchronization	↑ RPE ↓ distance covered high intensity ↑ distance covered moderate and low intensity ↑ acceleration and deceleration ↓ distance between dyads ↑ distance between dyads ↓ longitudinal synchronization
Barte and collaborators (2020)	To verify the effect of fatigue on decision making in soccer	30 experienced soccer players (20.3 ± 3.3 years)	▪ High intensity intermittent running exercise protocol (Russell, et al. 2011). ▪ Pass interception test; ▪ Sprint test	▪ HR ▪ RPE ▪ sprint capacity ▪ motivation ▪ number of interception attempts ▪ % of interception effect	↑ HR; ↑ RPE ↔ sprint capacity ↓ motivation to intercept the pass ↔ number of interception attempts ↔ % of correct answers for interceptions.

*↓* drop, ↑ increase (both with statistical significance); ↔ maintenance (without statistical significance); HR—heart rate; [la]—lactate concentration; RPE–rate of perception of effort; LSPT—Loughborough Soccer Passing Test.

## Discussion

This study aimed to carry out a systematic review in order to analyze, describe and discuss the effect of physical fatigue on the performance of soccer players. In this way, it is intended to show a compilation of the studies and discuss possibilities for investigation and intervention, considering physical fatigue as a conditioning factor in the cognitive, technical, physical and tactical performance of soccer players.

The articles identified in the present systematic review were classified as presenting high methodological quality (77.80%). In general, the quality analysis suggested that the aims of the studies were clearly stated, the scientific literature of the area was revised, and the ethical procedures required for studies with human beings were respected. Also, the statistical procedures and the variables analyzed were well defined in the studies. The main methodological deficiencies identified by the quality assessment were the lack of justification for the sample size, the lack of information regarding voluntary dropouts, and the non-inclusion or superficial presentation of the studies’ practical implications.

The results obtained in recent years in relation to the cognitive performance of soccer players in a state of physical fatigue have shown inconsistencies. On the other hand, studies have shown negative effects of physical fatigue on the players’ physical and technical performance. In addition, there is a lack of studies addressing the individual and collective tactical performance of players in a state of physical fatigue. In order to detail the work on the topic, in this review the attention will be directed in order to understand each session of the selected articles from a look at the topics: focus of the studies; sample description; analysis instruments and procedures; analysis of results and guidance for future work.

### Study focus

With regard to the objectives of the selected works, it is noted that out of a total of 12 articles that met the inclusion criteria, eight evaluated the effect of physical fatigue on performance, two evaluated the effect of intermittent physical exercise on performance and two evaluated the effect of physical exercise with fixed intensity on performance. In these studies, players were evaluated in two situations, one being control (e.g., without inducing physical fatigue) and the other experimental (e.g., with inducing physical fatigue), in order to verify how physical fatigue affects the performance of players of soccer.

Over the years, the objectives of the selected works have changed in direction. The first published works on the theme, between 1996 and 2016, sought to investigate the effects of physical fatigue on cognitive performance [32–34] and technical through a single perspective [35]. However, more recently, experimental studies have begun to investigate the effects of physical fatigue on performance more globally, involving analyses with more than one aspect, for example, perceptual-cognitive skills and motor aspects of movement and displacement [23,36,37]. In the context of soccer, despite the players’ actions being guided by tactical aspects aiming the occupation of the field and the management of the playing space [2], only a recent work developed by Coutinho and colleagues (2018) sought to investigate the effect of muscle fatigue on the teams’ collective tactical behavior. The evidence from this study allowed to identify a change in the collective tactical behavior under a state of muscle fatigue, which provided an important reflection in relation to the individual tactical behavior of the players, since the synchronization measures obtained by Coutinho and colleagues (2018) do not consider the result of individual tactical actions.

### Sample description

The selected studies showed considerable variability in relation to the sample size, with the average number of players used per study ranging from 10 to 30 players. Due to this variation, the comparison and generalization of the results of the studies included in this systematic review should be done with caution, given that small samples present limited representativeness in relation to the reality of the game context.

Still in relation to the samples of the articles in the present review, three articles set out to compare players of different levels, that is, experienced vs. inexperienced players and elite vs. sub-elite [32,34,38], one article evaluated professional players [39], three articles evaluated semi-professional players [37,40,41], four articles evaluated amateur players [23,33,36,42] and an article evaluated young soccer players [35]. Based on this, it is observed that the samples of the selected articles are heterogeneous, and that the effects of physical fatigue and physical exercise affect different populations of soccer players. In addition, it should be noted that studies have shown that players with a high level of experience tend to suffer less negative effects from physical fatigue than less experienced players, indicating the knowledge accumulated throughout the process as an intermediate variable under a state of physical fatigue. This can be explained by the players’ familiarity with the physical and metabolic demands of the task, and by the knowledge acquired throughout the formation/training process.

### Analyses instruments and procedures

The selected studies used different tasks to induce physical fatigue, with emphasis on: 1) treadmill protocol; 2) cycle ergometer protocol; 3) specific test (Léger, Loughborough Intermittent Shuttle Test–LIST, RCOD); 4) adapted specific physical test; and 5) game simulation. Simultaneously to the performance of the task, some studies adopted physical and physiological measures (Sprint, Distance covered, Heart Rate–HR, Subjective Perception of Effort–PSE, Lactate–La) as control variables to verify the effectiveness of the task in inducing physical fatigue as proposed.

The first studies on this theme identified in the present review indicated a trend towards the choice of physical tests with fixed intensities on a treadmill or cycle ergometer [33,34,38]. These tests proved to be capable of increasing the rates of subjective perception of effort, heart rate and physiological responses. However, the researchers found it difficult to explain the positive effects caused by these physical protocols on the players’ performance, especially at maximum intensities. Therefore, the most recent research in the area has been using physical protocols more applied to soccer, in order to simulate the physical demands of the game and the duration of the game [32,36,41]. Although they present an advance in relation to the applicability and representativeness of the soccer game, these protocols, for the most part, do not include actions of acceleration, deceleration, changes of direction, jumps and ball actions, which are present demands during the game and which generate an increase in the physical wear of the players [43]. Thus, it is important to pay attention to the particular characteristics of physical tests, since there is evidence that demonstrates that different physical protocols can modify the performance of players [26,44]. Thus, it is recommended that the use of physical protocols composed of more representative actions of the soccer game be considered in future investigations.

As for the assessment of the cognitive performance of the players, the use of tests of specific soccer videos stands out for the assessment of the variables decision making and anticipation [32,38]. On the other hand, other specific tasks were also employed in order to obtain measures related to the players’ decision making [34,37]. In addition, the multiple choice task test contained in the Vienna System was used to measure the reaction time of the players [33]. Although it is not an ecologically valid test, multiple choice tests require the use of relevant cognitive skills necessary for soccer performance (e.g., attention and perception). However, the specificity of the task seems to be an aspect that influences the cognitive results presented by players after physical protocol. Studies that used cognitive tasks with greater representation of the soccer game (video scenes) were more sensitive in identifying the effects of physical fatigue on the players’ cognitive performance [32,37,38]. Previous studies revealed that the effects of physical fatigue on cognitive performance are especially dependent on the types of tasks used, in addition to being more sensitive in characterizing the differences between groups of different levels of performance [26]. Therefore, the interpretation of these results can be centered on the methodological aspects of the studies.

Regarding technical performance, some articles used the Loughborough Soccer Passing Test (LSPT] and Loughborough Soccer Shooting Test (LSST) tests to assess the efficiency of the pass and the shooting, respectively, [35,40], while other studies used specific predetermined tasks designed to assess the technical basis of the pass [39], shooting [39,42] and interception [36]. Regarding the dynamics of the pass test, players should execute 16 passes in the shortest possible time on benches positioned around them, with each mistake made, players were penalized with an increase in time on the task. On the other hand, in the shooting test, players should perform movements such as accelerations, changes of direction, ball control and finishing on goal while being assessed for accuracy of the shooting and the speed of the ball in finishing. In the interception test, the players had 15 attempts to intercept the pass. In each attempt, if the player thought he would be able to intercept the pass, the player should run towards the ball and perform the interception before it reaches a predetermined target. Otherwise, the player should run in the opposite direction. It should be noted that no work identified in the present systematic review evaluated the technical performance of the players during the game.

In order to measure tactical performance, the collective tactical behaviour was assessed based on longitudinal/lateral synchronization measures and the team’s contraction speed during reduced games [23]. In addition, physical measures of distance covered, acceleration and speed were also evaluated. It is worth mentioning that the GPS was the instrument used to collect the physical and tactical information of the players, which has been showing itself as an important resource for assessing the physical and tactical variables in the context of the game.

In general, studies in this area showed an advance in methodological aspects in relation to physical protocols, as well as in the tests adopted in the performance evaluation. However, the aforementioned instruments, equipment and tests still have low ecological validity, which is an important aspect to be considered in future investigations.

### Analysis results

The main results found in the selected studies in relation to the cognitive, technical, physical and tactical performance of soccer players will be highlighted.

#### Cognitive performance

In general, the studies that investigated the cognitive aspect showed divergent results about accuracy. The cognitive response tends to vary according to the cognitive task employed and the physical protocol used. Despite that, most studies included in this systematic review indicated that speed/response time of cognitive tasks tends to improve after a situation of physical effort.

Among the studies, methodological differences were observed with regard to the physical protocol used and the task of evaluating decision making [34,38]. In the article by McMorris and Graydon (1996), the authors used a protocol in which the image of an offensive scene in situation 6 vs. 6 was projected on a screen and the evaluated player should select the most appropriate action by the ball carrier (run with the ball, shooting, pass or try the dribble). In turn, Fontana and colleagues (2009) evaluated decision-making from offensive scenes from a third-person perspective, which provided a view of the entire playing field. In this case, the evaluated player should choose the most appropriate action, among four pre-defined options, for the ball carrier. The studies also used different equipment for the tests of physical effort induction, being the cycle ergometer [34] and the treadmill [38].

Based on these results, the declarative knowledge of soccer players seems that was not affected by physical fatigue since the players were able to find the best options for the situations presented on the tests regardless of their physical condition. However, the researchers should be cautious in interpretating these results, especially linking physical fatigue with improvements in decision-making (maintenance of the quality of decision-making and a decrease in the response time). In that sense, more studies need to be performed to verify this relationship and also analyze if other variables could have been influencing the results, for example the working memory, characteristic of the test, or type of physical protocol. It is important to emphasize that the psychobiological model of fatigue, which considers the role of effort on players’ responses, can contribute to interpreting the results of the decision-making of soccer players under physical fatigue. This model assumes that the conscious regulation (decision-making) of the exercise’s pace is determined by motivation and, above all, by the player’s perception of effort [45]. In this conjecture, the motivational status of the players can be a factor that influences their decision-making under physical fatigue, which may contribute to the observed reduction of the time required by the players to complete the task while keeping the precision of the decision. In this regard, future investigations should also consider these psychobiological aspects and apply more ecologically validated decision-making tasks.

On the other hand, Casanova and colleagues (2013) showed that intermittent physical exercise negatively impacts the perception, information processing and anticipation processes of elite and sub-elite players. However, the drop in performance observed for the elite group was smaller compared to the sub-elite group. Elite soccer players have also undergone minor changes in the visual search pattern, in addition to adapting it to extract the necessary information even in situations of high physical demand. These results suggest that intermittent physical exercise has negative effects on the perceptual-cognitive processes of soccer players, which may lead the player to make worse decisions during the game and, consequently, present a drop in tactical efficiency, which differs from the results presented by McMorris and Graydon (1996) and Fontana (2009).

Regarding perception, although a specific test for soccer was not used, the study conducted by Lemmink and Visscher (2005) [33] concluded that there were no differences in reaction time, correct, incorrect and omitted reactions when comparing visual responses of players between the control (without physical effort) and experimental (submitted to intermittent physical exercise) groups. However, the results demonstrated an improvement in the reaction time and an increase in the number of correct reactions when the responses were compared between the blocks of the physical intervention protocol in the experimental group. These results suggest that this improvement may be related to the characteristics of the physical protocol, which may have generated greater excitement of neural aspects and promoted the accumulation of neurotransmitters in the brain [46]. A similar explanation has been discussed in studies that show a positive relation between exercise and response/reaction time in simple and choice reaction time tasks, meaning that the responses are faster during the exercise [26].

In particular, this improvement could be due to an unconscious compensation of other aspects of the game that were impaired, such as the physical aspect [47]. In sports like soccer, it is important to efficiently select the right action at the right moment during a game [48]. Many studies have indicated that achieving a high-level performance requires not only accurate execution of motor behavior but also consistent perceptual-cognitive skills [49,50]. Therefore, this finding is interesting since well-develop perceptual-cognitive skills could be one aspect to help player sustain their performance during a situation of fatigue.

Finally, the study by Frýbort and colleagues (2016) found an increase in the response time aimed at the motor (perceiving, processing the information, making the decision and performing the motor action) after physical fatigue, although the ability to choose the correct option, that is, the quality of decision-making has not been changed. The increase in the visual-motor response time can be attributed to the accumulation of energetic substrate (k^+^, I^+^, lactate and creatine kinase–CK) during the physical protocol and to the loss in the efficiency of the neuromuscular recruitment mechanism, which, consequently, impairs biomechanics of the technical gesture, generating an increase in the time for the execution of the action [14].

#### Technical performance

Concerning the technical performance, the results suggested that physical fatigue negatively affects the technical fundamentals of the pass, dribble and kick. These actions present a relationship with motor movement. To date, research in this area have indicated that physical fatigue exerts a negative influence on the power output of leg muscles and movement biomechanics [51,52]. In this regard, technical fundamentals that depend on movement precision and efficiency tend to be negatively affected by physical fatigue.

Regarding the technical basis of the pass, Russell, Benton and Kingsley (2011) observed a decrease in the accuracy of this technical gesture. Likewise, Rampinini and colleagues (2008) also found negative effects of physical intervention in relation to the pass, with a decrease in the accuracy of the short pass, and an increase in penalties due to mistakes committed. In the same study, the authors also found a negative correlation between the distance covered on the Yo-Yo Intermittent Recovery Test Level 1 and the accuracy of the pass, indicating that the increase in distance covered on the Yo-Yo Intermittent Recovery Test Level 1 was detrimental to performance technician. These results corroborate with other studies that have demonstrated that actions involving movement and required motor adjustments are negatively affected by physical fatigue (Frýbort et al., 2016). Overall, both studies evaluated the technical fundamentals of the pass using pre-defined tasks under time pressure and adopted precision as the evaluation criteria. However, the effect of physical fatigue on this fundamental was not analyzed during game situations, in which the players are free to choose when and how it is more appropriate to perform the pass. In this regard, it would be interesting for future studies to evaluate the velocity of transmission of the ball (an index calculated through the division of the number of passes by the number of contacts with the ball). The analysis of this metric could be used to identify the game intensity, given that the closer the value is to one, the greater the game intensity tends to be. This analysis could advance the literature and improve the understanding of how players under physical fatigue manage their actions when in possession of the ball.

As for dribbling performance, the study by Stone and Oliver (2009) showed that players submitted to 45 minutes of physical protocol took longer to complete the task of dribbling. On the other hand, Russell, Benton and Kingsley (2011) submitted players to a simulation protocol of the soccer game, lasting 90 minutes, and verified the maintenance of accuracy, percentage of correctness and the average dribbling speed of the players after the physical protocol. The dynamic of the soccer game allows players to self-control their pace [53,54]. Then, in situations of acute physical fatigue or during the last stage of the game, players tend to reduce the pace as a strategy to maintain performance [23]. This premise seems to occur for dribbling, mainly in time/penalty tasks like the studies used to investigate this tactical aspect. Then, during a section of training or over the game, coaches could search for activities and strategies that promote, short pass, prioritizing possession of the ball, and avoiding situations of confronting 1 vs. 1 since they enhance the chance of loss of the ball.

In addition, the study by Barte and colleagues (2020) observed that the technical basis of the interception of the pass does not depend on the physical and physiological state of the player, since there was no change in the number of attempts and the percentage of correct answers of interceptions after inducing physical fatigue. However, the players felt less motivated to perform the interception action when subjected to physical fatigue. This lack of motivation may be linked to the decisions taken during the soccer game to be influenced by individual and contextual factors [55,56], in addition to motivational and emotional factors [57]. In this scenario, the findings of the study conducted by Barte and colleagues (2020), seem to occur because, despite the cognitive task used involves physical, cognitive and technical aspects, there is no presence of contextual factors, which may have limited the motivation of players to perform the task.

Other research has proposed to investigate the effect of a physical protocol on the technical basis of the shooting [40,42]. The study by Stone and Oliver (2009) found that the players showed a decrease in the accuracy of the shooting after physical fatigue. Still in this context, the study by Ferraz, Tillar and Marques (2012) observed a reduction in the average and maximum shooting speeds. The loss in the quality of the shot may suggest that the player will find it more difficult to frame the shot on goal and, consequently, score the goal, after a high demand for physical effort during the game.

Based on this, although the increase in the frequency of goals in the final moments of the match has been linked to the physical wear of the players [25], other aspects may also be related to this increase, such as mental fatigue, a feeling of tiredness and lack of energy experienced during the game due to the numerous decision-making situations [48]. Studies show that players under mental fatigue make more tactical mistakes and make worse decisions [47,58]. Thus, when we consider that in the defensive phase, players are under constant pressure not to make mistakes, since making mistakes in this phase can mean loss of organization and defensive security and an increase in the chances of goal shooting by the opposing team, it seems plausible to infer that mental fatigue can also be considered as a possible factor influencing the players’ performance. Therefore, in practical terms, the frequency of goals conceded in the final moments can be pointed out as a result of multifactorial aspects.

#### Physical and tactical performance

As for physical performance, the negative impact of physical fatigue on the players’ ability to perform sprints is highlighted [39,41]. In addition, in the study conducted by Small and colleagues [41], players in situations of physical fatigue showed changes in the biomechanical pattern of sprint movement and, consequently, an increase in the risk of hamstring injury. On the other hand, in a game situation, there was an increase in acceleration actions and in distances covered at low and moderate intensities, in addition to a reduction in distances covered at high intensity [23]. This incongruity of the results seems to occur due to the dynamics of the reduced games, which provide greater freedom to the players to adjust their efforts and modify the pace of the game. In game situations, when the perception of effort is increased due to high physical effort, the player tends to alter or adapt his displacement and movement rhythm, in order to avoid falls in technical and cognitive performances [45,59].

This finding is also evidenced by the study by Coutinho and colleagues (2018), which investigated the effects of physical fatigue on collective tactical behavior in soccer. In specific situations of reduced game, there was an increase in synchronization between the players. In general, the players started to act more compactly. In relation to the game, this greater proximity between the players means a decrease in the effective playing space, less dispersion of the players on the field and a more stable collective behavior. These results were attributed to a collective strategy to overcome the negative effects of physical fatigue on the intensity of movement and displacement of the team. As in soccer the players perform physical efforts for 90 minutes, constantly varying in intensity throughout the game [60], physical fatigue and, consequently, its effects appear to be momentary during the game. Thus, the degree of synchrony presented between the players of the teams should not necessarily be interpreted exclusively as tactical behavior only in the final moments of the match, since they can also occur occasionally throughout the match.

### Study limitations, future research and clinical implications

Due to the lack of studies conducted with similar approaches, it was difficult to provide solid evidence about the effect of physical fatigue on players’ performance, mainly with regard the cognitive and tactical aspects. Overall, the cognitive tests used showed low representativeness with the soccer game. In addition, there was a lack of studies that aimed to investigate the effects of physical fatigue on the collective and individual tactical performances of soccer players. Finally, the metabolic stimuli of the majority of the physical protocols used were distant from the physical and metabolic demands of the soccer game. The sum of these aspects can influence the results found, besides hindering their interpretation and, consequently, impairing the application of this knowledge in the practical context of the soccer game.

In view of these limitations, it is necessary that future studies seek to use protocols for inducing physical fatigue with metabolic stimuli similar to the physical and physiological demands of the soccer game, and which use more representative evaluative tasks (*in vivo* or *in vitro*) in relation to the context of the modality. In addition, sports performance should not be analyzed in isolation, as the soccer game has a dynamic nature and presents a series of factors that emerge and interrelate during the match. It is therefore suggested that future studies use a more holistic approach, proposing to analyze the effect of physical fatigue on cognitive, tactical, technical and physical aspects, in order to broaden the horizon of knowledge on this topic. The understanding of this relationship can positively impact the planning of training activities for the development of the players, aiming at the conformity of the content with the specific demands of the game.

## Conclusions

According to the results discussed in this systematic review, it is possible to conclude that physical fatigue has a negative effect on the individual performance of soccer players. However, a large part of the studies discussed here presented methodological differences, such as the use of different protocols for inducing physical fatigue and the use of different performance assessment tasks (tactical, cognitive, technical and physical). It is also important to highlight that many of the protocols and tasks used by the studies are far from the reality of the soccer game, a fact that must be considered in the interpretation of these results.

## Supporting information

S1 Checklist (PDF)
